# Editorial: Recent advances in recognition of bioactive phytonutrients for specific targets in plant foods

**DOI:** 10.3389/fnut.2022.1018946

**Published:** 2022-10-03

**Authors:** Zhiqiang Wang, Mingquan Guo, Soon Sung Lim

**Affiliations:** ^1^Key Laboratory of Public Health Safety of Hebei Province, School of Public Health, Hebei University, Baoding, China; ^2^Key Laboratory of Plant Germplasm Enhancement and Specialty Agriculture, Wuhan Botanical Garden, Chinese Academy of Sciences, Wuhan, China; ^3^Department of Food Science and Nutrition, Hallym University, Chuncheon, South Korea

**Keywords:** phytonutrients, new analytical methods, target-based recognition, metabolomics, biological activity

Changes in lifestyle have resulted in an increased susceptibility to aging and various life-threatening diseases, such as cancers, coronary heart disease, obesity, and diabetes mellitus. Plant-based foods have received much attention in recent decades, as they provide major nutrients and are indispensable sources of bioactive phytonutrients, which have long been shown to be beneficial in preventing aging and the aforementioned diseases (1). Many phytonutrients interact with the biologically active sites of target proteins that are involved in the pathologies of aging and associated diseases. Therefore, there is an increase in research interest in the exploration of non-nutritive bioactive components in plant foods, especially the identification of phytonutrients with specific targets that facilitate the characterization of their chemical diversity and pharmacological activities (2). This Research Topic highlights recent advances in the identification of bioactive phytonutrients, which have long been known to exist in plant-based foods, but their specific protein targets have remained elusive.

Diabetes mellitus is a metabolic disorder of the endocrine system characterized by hyperglycemia. It has become one of the most intractable global public health challenges (3). Plant-based foods, as source of a diverse range of phytonutrients, are considered potential therapeutics for diabetes. They exhibit multiple therapeutic effects on the pathologies of abnormal glucose and lipid metabolism, and possess beneficial antioxidant activity. Consequently, Xie et al. reported the *in vitro*/*in vivo* hypoglycemic and antioxidant activities of the *Amomum taso-ko* fruit; Huang et al. reported the effects of red ginseng on lipid metabolism in an animal model of type 2 diabetes mellitus, and Wang, Tong et al. reported the hypoglycemic activity and associated mechanisms of ginseng berry using ultra high performance liquid chromatography (LC)-mass spectrometry (MS)-based metabolomics. Moreover, the identification of bioactive phytonutrients from *Amomum taso-ko* fruit, red ginseng, and ginseng berry, which may contribute to the hypoglycemic activity, was performed using high-resolution LC-MS/MS. Based on their results, LC-MS/MS, as an important untargeted metabolite profiling tool, is a powerful technique for the identification of bioactive phytonutrients in plant-based foods.

The isolation of bioactive phytonutrients is a well-established and non-substitutable approach that relies on subjecting mixtures of phytonutrients to iterative steps of fractionation and biological testing. The underlying strategy is aimed at reducing the complexity of plant-based food composition until a group of phytonutrients or a single phytonutrient with specific biological activity is obtained, and the structures and biological activity of the purified phytonutrients can be fully characterized. In this context, the selection of reliable targets or model assays for the assessment of biological activity is of great concern. For example, pancreatic lipase inhibitor therapy is an effective method of preventing and treating obesity. In the past few decades, porcine pancreatic lipase has been widely used for screening pancreatic lipase inhibitors. In contrast, efficacious inhibitors of human pancreatic lipase have rarely been reported. Although the amino acid sequence identity between porcine and human pancreatic lipases is relatively high (~86%), remarkable intra-species differences in inhibitor responses have been reported (4). Herein, the establishment of a fluorescence-based high-throughput assay is reported by Qin et al.. In this assay, human pancreatic lipase was used as the specific target and a near-infrared fluorogenic substrate was used as the probe. Phytonutrients with activity against human pancreatic lipase were identified after their isolation from *Ampelopsis grossedentata*.

However, the isolation process has also been criticized for real and perceived weaknesses, such as being time-consuming and labor-intensive, and the ease by which trace phytonutrients are lost. Therefore, with progress in the development of new technologies and materials in the field of analytical chemistry, new strategies for the *in situ* identification of bioactive phytonutrients for specific targets in plant-based foods, without the requirement for tedious purification processes, are being developed. In particular, magnetic-ligand fishing is a feasible strategy. To reduce the false-positive rate in ligand fishing due to the non-specific binding of immobilized enzymes, Chi et al. prepared and applied GO@Fe3O4@SiO2-COX-2 nanoparticles to fishing ligands of cyclooxygenase-2 in *Choerospondias axillaris*, since graphene oxide maintains the ligand structure and physiological activity when combined with protein targets. Microfluidic platforms are versatile tools for screening bioactive molecules from plant-based foods. A rapid microfluidic chip-based ligand fishing platform for discovering matrix metalloproteinase-2 inhibitors from *Rosa roxburghii* was developed by Tao et al.. They used a layer-by-layer assembly approach to fabricate core-shell CdSSe@ZnS-quantum-dot-encoded superparamagnetic iron oxide microspheres to serve as a carrier for matrix metalloproteinase-2. Affinity-based ultrafiltration combined with LC-MS/MS is another feasible strategy for the *in situ* identification of bioactive compounds in plant-based foods. Herein, the investigation of potential anti-aging components of *Moringa oleifera* leaves is reported by Xu et al., who used affinity-based ultrafiltration with multiple drug targets. Meanwhile, a combination of high-speed counter-current chromatography, affinity-based ultrafiltration, and LC-MS/MS was reported by Wang, Wang et al. for the *in situ* identification of minor tyrosinase inhibitors from *Dryopteris crassirhizoma* rhizomes. Based on these results, affinity-based techniques, combined with modern analytical methods, provide a solution for the rapid identification of bioactive phytonutrients with specific targets in plant-based foods, without the need for further time-consuming and labor-intensive isolation and purification steps.

However, the specific targets of the identified phytonutrients should be explored and validated using molecular mechanisms. For example, (-)-epigallocatechin-3-gallate (EGCG) has been shown to be involved in the proliferation and apoptosis of human nasopharyngeal carcinoma (NPC) cells; however, little is known about the potential targets of EGCG in NPC cells. Jiang et al. reported that EGCG inhibits cell proliferation and induces apoptosis by downregulating SIRT1 expression levels. In another case, the adjuvant potential of lycopene against the metastasis of lung cancer cells was reported by Chan et al.. The results of their study suggested that lycopene and sorafenib additively inhibit the mitogen-activated protein kinase pathway by reducing the phosphorylation of ERK1/2, JNK1/2, and p38.

In conclusion, the present Research Topic provides several examples of advanced techniques used for the screening, identification, and subsequent investigations of the mechanisms of action of bioactive phytonutrients with specific protein targets, in plant-based foods. Moreover, this collection will promote further research and development activities on the plant-based foods described in this issue, and provide new ideas and techniques for the further development and utilization of other plant-based foods.

## Author contributions

ZW drafted the manuscript. MG and SL provided critical review and insight and revised the final version of the editorial. All authors contributed to the article and approved the submitted version.

## Funding

The financial support for this work was provided by National Natural Science Foundation of China (81803401) and Natural Science Foundation of Hebei Province (H2022201021).

## Conflict of interest

The authors declare that the research was conducted in the absence of any commercial or financial relationships that could be construed as a potential conflict of interest.

## Publisher's note

All claims expressed in this article are solely those of the authors and do not necessarily represent those of their affiliated organizations, or those of the publisher, the editors and the reviewers. Any product that may be evaluated in this article, or claim that may be made by its manufacturer, is not guaranteed or endorsed by the publisher.
